# Entomopathogenic Fungi Biodiversity in the Soil of Three Provinces Located in Southwest China and First Approach to Evaluate Their Biocontrol Potential

**DOI:** 10.3390/jof7110984

**Published:** 2021-11-18

**Authors:** Wei Chen, Weiwen Xie, Wei Cai, Narit Thaochan, Qiongbo Hu

**Affiliations:** 1 Key Laboratory of Bio-Pesticide Innovation and Application of Guangdong Province, College of Plant Protection, South China Agricultural University, Guangzhou 510642, China; cw@stu.scau.edu.cn (W.C.); weiwenxieGZ@163.com (W.X.); SCAUcaiw@163.com (W.C.); 2 Agricultural Innovation and Management Division (Pest Management), Faculty of Natural Resources, Prince of Songkla University, Hat Yai 90110, Songkhla, Thailand; narit.t@psu.ac.th

**Keywords:** insect control, species diversity, *Bemisia tabaci*, *Spodoptera litura*

## Abstract

Entomopathogenic fungi (EF), who represent active agents to control insect natural populations, usually persist in terrestrial habitats. Southwest area in China has various climate conditions and abundant plant biodiversity (crop, forest, grassy, orchard and arable areas). Nevertheless, the potential of soil-inhabitant EF as insect pest biocontrol agents, is unknown. In this study, first the EF biodiversity from soil of three provinces (Guizhou, Sichuan, and Yunnan) was surveyed. Then, the virulence of 29 isolated strains against *Bemesia tabaci* and *Spodoptera litura* was assessed. After analyzing 212 soil samples, 497 isolated fungi were identified. Out of them, 490 isolates were classified in 45 species of 24 genera, whereas the other seven isolates, belonging to *Paecilomyces* and *Purpureocillium* genera, were not identified under species level. Furthermore, the EF biodiversity from soil of Sichuan, Yunan, and Guizhou areas, analyzed by Shannon Wiener Index (SWI) was rated at 2.98, 1.89, and 2.14, while SWIs-biodiversity in crop, forest, grassy, orchard and arable areas was rated at 2.88, 2.74, 3.05, 2.39, and 2.47. SWI data suggested that soil from Sichuan area and grassy had higher EF biodiversity compared with other analyzed provinces and areas. Virulence bioassay results indicated that, out of the 29 isolates tested, 24 were pathogenic against *B. tabaci* and *S. litura*, resulting in mortality rates >10%. In conclusion, this study reports the EF distribution and biodiversity in soil from three provinces located at Southwest China, whereas their potential use as a tool for the *B. tabaci* and *S. litura* biocontrol must be further investigated.

## 1. Introduction

Entomopathogenic fungi (EF) play an important role in pest biocontrol and have high economic significance. There are more than 1000 EF species in 100 genera recorded in the world [1]. The most commercialized EF species, *Beauveria bassiana* and *Metarhizium anisopliae*, have been extensively developed as mycoinsecticides in China (China Pesticide Information Network. Available online: http://www.chinapesticide.org.cn/hysj/index.jhtml, accessed on 7 April 2021) and other countries [2,3,4,5]. In addition, *Isaria fumosorosea* (*Paecilomyces fumosoroseus*), *Lecanicillium lecanii* (*Verticillium lecanii*) and *Purpureocillium lilacinum* (*Paecilomyces lilacinus*) are often used for pest control worldwide [6].

EF have complicated life cycles. The most hypocreales EF usually have two stages, the infecting stage occurs on host insects during from adhering of conidia on cuticle to production of new conidia on insect cadavers. In the second stage, EF persist in soil and live in rhizosphere or grows as endophyte [7]. In the life cycle, a great number of conidia fall into soil from insect cadaver died by EF. Therefore, soil is an important shelter of EF and a pathogen pool of insects as well. However, soil environments undoubtedly are influenced by the human activities under rapid development of society and economy, and impacted by the constantly climatic changes [8]. These factors could affect the EFs distribution and persistence in soil [9,10]. Therefore, in order to determine the soil EF potential as mycopesticides, it is necessary fist to analyze their biodiversity, to identify the persistent species and then to bioassay their virulence against insect pests of agricultural importance.

Both the *Bemesia tabaci* and *Spodoptera litura* are worldwide pests that have caused huge economic losses to agriculture worldwide [11,12]. Currently, the control of both *B. tabaci* and *S. litura* relies mainly on chemical pesticides, but the massive use of chemical pesticides has led to an increasingly serious problem of pest resistance and serious environmental pollution. Therefore, biological control methods are receiving increasing attention. EF are important biological control resources, which are selective, harmless to humans and animals, have long residual effect period, remarkable prevalence, and are not easily resistant to pests, etc. They occupy an important position in biological control of pests [13]. It is of great interest to screen for strains with high potential pathogenicity against *B. tabaci* and *S. litura*. Southwest China is one of areas with the most abundant biodiversity, depending on the distinct geography conditions with the Yunnan-Guizhou Plateau, Hengduan Mountains and Sichuan Basin, and the various climates from tropical to cold zone. A lot of rare species of insects and other organisms are distributed in this area [14,15]. However, there are rarely reports about the soil microorganisms in this area. Obviously, investigation of soil EF not only to improve the soil microbiota biodiversity knowledge but also provide the resources of EF for biocontrol potential.

The current study was aimed to investigate the distribution and abundance of EF under different soil habitats in Southwest China, including Sichuan, Yunnan and Guizhou provinces, in order to determine the diversity and prevalence of EF in Southwest China, and provide new fungal resources for the biological control.

## 2. Materials and Methods

### 2.1. Soil Sample Collection

**Figure 1:**
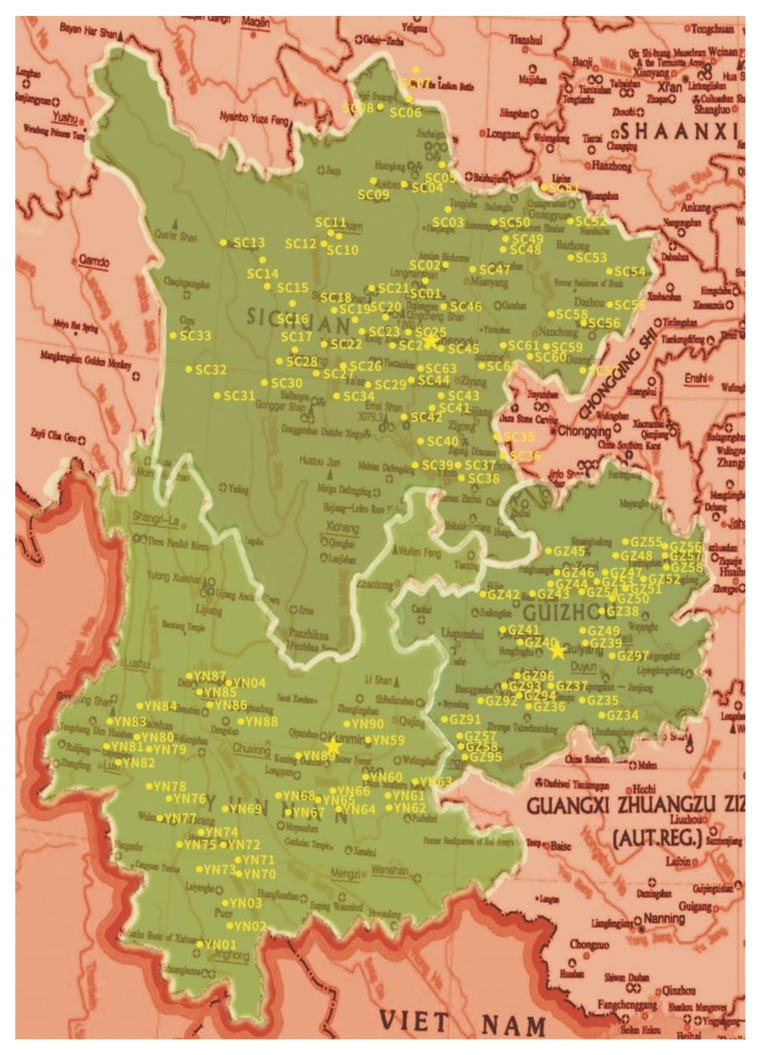
The map of sites distribution for the soil samples collection.

Soil samples were collected in different habitats, including crop, forest, grassy, orchard and arable. The longitude and latitude in each site were recorded by ICEGPS 100C (Shenzhen, China). From each site, approximately 100 g soil beneath the ground 10–15 cm in three randomly selected points were collected and mixed as a sample stored in a plastic bag at 4 °C for further use. The total 212 samples were collected from 133 sites in three provinces, Sichuan, Yunnan and Guizhou of Southwest China (Figure 1).

### 2.2. Isolation of Fungi from the Soil Samples

First, let each soil sample pass through a 40 mesh sieve (425 micron aperture) and separate into three batches of 10 g. Each batch was suspended with 100 mL 0.1% Tween-80 solution. Then, 100 μL suspension from each batch was inoculated on the selective medium (potato 200 g/L, glucose 20 g/L, agar 20 g/L, 50 mg/mL actinomycin 4 mL/L, 50 mg/mL chloramphenicol 4 mL/L, Bengal red 0.013 g/L) and cultured on 25 ± 1 °C. When fungi grow out, the single colony was transferred on PDA (potato 200 g/L, glucose 20 g/L, agar 20 g/L) plate and cultured at 25 ± 1 °C for identification [16]. The isolates were maintained with PDA slopes and sand tubes.

### 2.3. Identification of Fungal Isolates

The fungal isolates were identified based on the morphology and similarity of the rDNA-ITS sequences. In general, the colonies features on PDA plates were surveyed, while the conidia and sporulation structures were measured by optical microscope system equipped with a digital camera (MC-D500U, Phenix, Jiangxi province, China). For ITS sequence analysis, the total DNA from each isolate was extracted by using the DNA extraction kits (DP3112, Bio-Teke, Beijing, China) and referring to its protocol. The sequences were amplified by employing a T100TM Thermal Cycler (BIO-RAD, Berkeley, CA, USA) with the primers ITS1 (5′-TCCGTAGGTGAACCTGCGG-3′) and ITS4 (5′-TCCTCCGCTTATTGATATGC-3′) and the standard PCR cycling protocol. After PCR products were sequenced, the ITS sequences were compared and analyzed by BLAST of NCBI and found out the fungal species with the highest sequence similarity. Then, the phylogenetic trees were constructed by MEGA-X with a statistical method of maximum likelihood, a bootstrap test of 500 replications, and the Jukes–Cantor model [17]. ITSs of types from NCBI taxonomy were referred.

### 2.4. Evaluation of Shannon–Wiener Index

The biodiversity of fungal species was evaluated by the Shannon–Wiener index (SWI). SWIs were calculated based on the Formula (1).

(1) $$H=-\sum_{i}^{s}\left(\mathrm{Pi})(\mathrm{lnPi}\right),$$

where H is the value of SWI; s is the total number of species; i is the number of individuals of species; Pi is the proportion of the species i in the total number; lnPi is the value of the natural logarithm of Pi.

### 2.5. Bioassay of Fungal Strains on B-Biotype Whitefly and Spodoptera litura

The spores of rare fungal isolates were collected from the PDA plates and suspended with 0.05% Tween-80 solution and calibrated to a stock of 1.0 × 10^8^ spores/mL. The working suspensions for bioassay were prepared by diluting the stock.

In the whitefly bioassay, the population of B-biotype *B. tabaci* reared with *Hibiscus rosa-sinensis* for more than 20 generations in a greenhouse was used. The leaf immersion method (referring to China agricultural standard NY/T 1154.14-2008) was employed. *H. rosa-sinensis* leaves with second instar nymphs were dipped into working suspension for 20 s during treatment. Treated larvae were reared using fresh *H. rosa-sinensis* leaves. The pest’s numbers were surveyed every 24 h after treatment. The nymphs were considered as diseased death when they lost their normal yellow-green color, turgidity, smooth cuticle structure, and subsequently mildew grown.

In the *S. litura* bioassay, the population was fed with a semi-artificial diet [18]. The treatments were immersion method (referring to China agricultural standard NY/T 1154.6-2006). Put the second instar nymph of *S. litura* into a 1.5 mL centrifuge tube and add 1 mL of the prepared spore stock solution, quickly covered, and reversed for 20 s, transferred to a disposable plastic bowl covered with filter paper, fed with special feed, placed at 25 ± 1 °C, photoperiod 14L:10D, relative humidity 75 ± 5% in an artificial climate chamber. The pest’s numbers were surveyed every 24 h after treatment. According to the characteristic hyphae growing on the surface of the insect’s body and observed the conidia and conidiophores to determine whether it was caused by fungal infection. The 0.02% Tween-80 solution was used as a control group. The experiment was replicated three times. Corrected mortality was calculated based on the Formulas (2) and (3).

(2) M=100%×a/b,

(3) C =100% × (d−e)/(1−e),

where M is the mortality; a is the number of number of dead nymphs; b is the number of the test nymphs; C is the corrected mortality; d is the Treatment group mortality; e is the control group mortality.

### 2.6. Statistical Analysis

All data were statistically analyzed using Excel 2010 and DPS 9.5 (Data Processing System, Zhejiang, China). The one-way ANOVA was performed as per Duncan’s multiple range test to determine the significant difference at *p* < 0.05.

## 3. Results

### 3.1. Entomopathogenic Fungi Species Diversity in Soils of Southwest China

**Figure 2:**
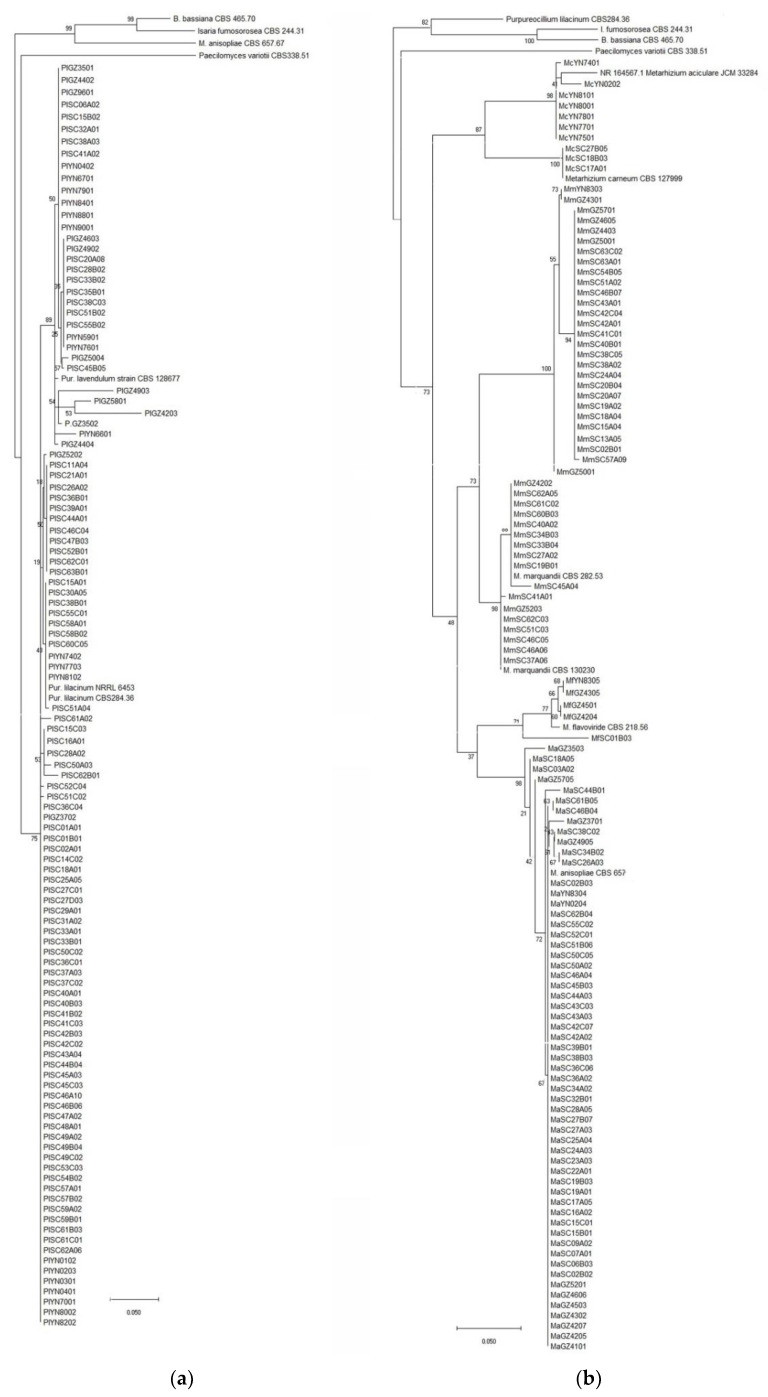
(**a**) Phylogenetic tree of *Purpureocillium* spp.; (**b**) Phylogenetic tree of *Metarhizium* spp.

**Figure 3:**
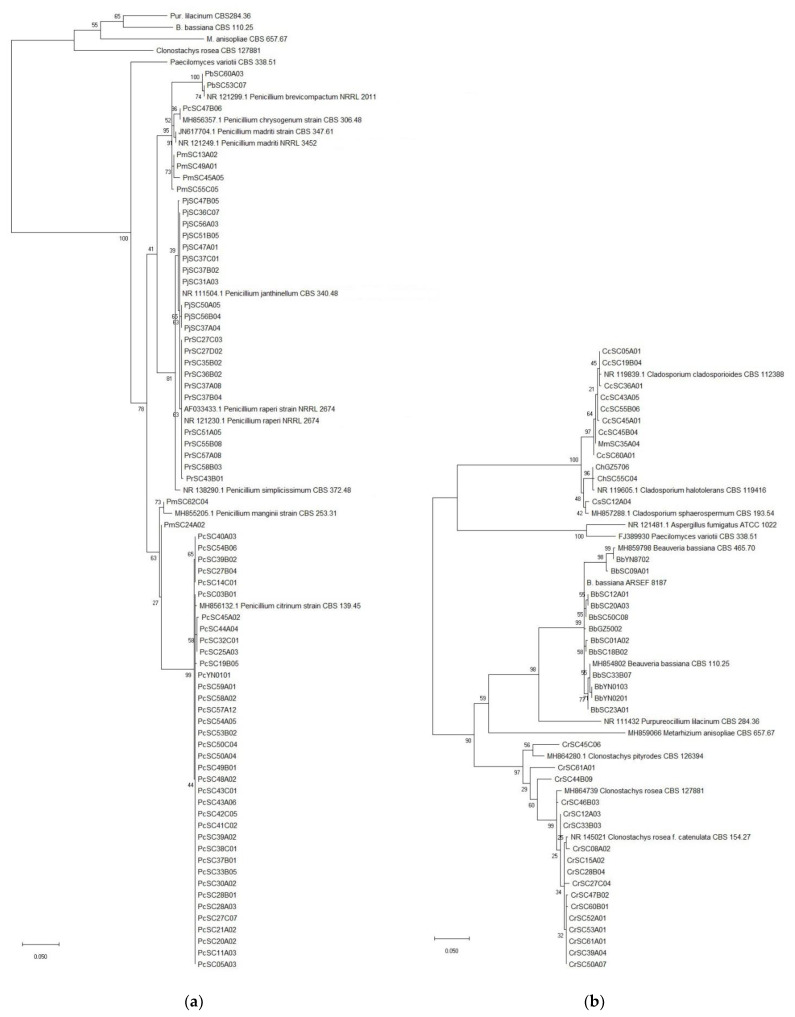
(**a**) Phylogenetic tree of the *Penicillium* spp.; (**b**) Phylogenetic tree of *Beauveria/Cladosporium/Clonostachys* spp.

**Figure 4:**
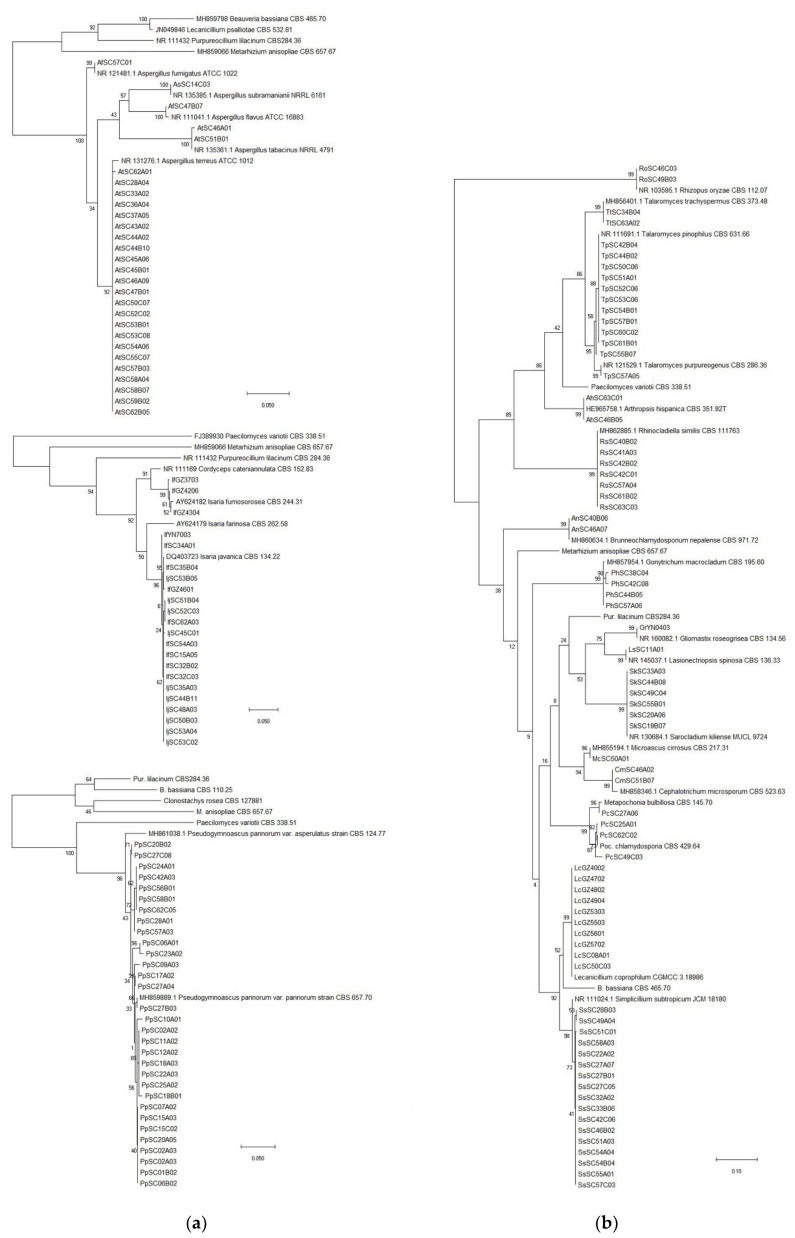
(**a**) Phylogenetic tree of the *Aspergillus*, *Isaria* and *Pseudogymnoascus* spp.; (**b**) Phylogenetic tree of other isolates.

A total of 497 fungal isolates were isolated from 212 soil samples, which 490 isolates in 45 species of 24 genera were identified based on the morphological and ITS analysis, while the other 7 isolates were not classified yet. Among them, 459 isolates of 32 species in 17 genera were reported as EF (Table 1). *P. lilacinum* with 82 isolates was the richest species, but the congeneric species *P. lavendulum* only had 30 isolates (Table 1, Figure 2a, Table S1). The genus *Metarhizium* with 5 species including *M. aciculare*, *M. anisopliae*, *M. carneum*, *M. flavoviride* and *M. marquandii*, respectively, had 7, 58, 3, 5 and 44 isolates (Table 1, Figure 2b, Table S1). The genus *Penicillium* had 5 EF species, *P. brevicompactum*, *P. chrysogenum*, *P. citrinum*, *P. Janthinellum* and *P. raperi*, respectively with 2, 1, 37, 11 and 11 isolates (Table 1, Figure 3a, Table S1). The genus *Aspergillus* had 3 EF species including, *A. flavus*, *A. fumigatus* and *A. terreus*, with 1, 1 and 23 isolates (Figure 4a, Table S1). While the other 13 genera each had 1 EF species with 1–30 isolates (Table 1, Figure 3 and Figure 4, Table S1). Obviously, *P. lilacinum*, *M. anisopliae*, *M. marquandii* and *P. citrinum* were the most abundant EF species.

### 3.2. The Distribution of Soil EF in Different Areas of Southwest China

The number of isolates and the rate of isolation of fungi and EF from different regions of Southwest China varied. Sichuan had 120 samples (all 124 soil samples) with fungi and EF, up to a 96.77% isolation rate and a 2.98 SWI (Table 2). Meanwhile, Yunnan and Guizhou had lower EF isolation rates of 67.50 and 70.83, as well as lower SWI values of 1.89 and 2.14 (Table 2).

### 3.3. The Biodiversity of EF in Different Soil Environments

The soil environment has a strong influence on the number and isolation rate of fungal isolates. The orchard and fallow soil samples had the higher EF isolation rates with >90%, followed by grassy and crop samples with 85–88%, and the lowest were forest samples with 73.17% only. However, the SWI indicated a different trend, which grassy soil had the highest SWI of 3.05 while fallow had the lowest SWI of 2.39 (Table 3).

### 3.4. The Pathogenicities of Fungal Isolates against B. tabaci and S. litura

A total of 29 isolates were subjected to bioassay on *B. tabaci* and *S. litura*. The results indicated that, when treated at the concentration of 1 × 10^8^ spores/mL, all isolates had a certain pathogenicity to whiteflies with a corrected mortality of 4–58%, except for *C. rossmaniae* CrSC40B04 (Table 4). The isolates IjSC62A03 of *I. javanica* had the best activity, with a corrected mortality of 57.78%. The species with no reported as EF, *A. subramanianii*, *A. tabacinus*, *A. hispanica*, *C. microsporum*, *C. halotolerans*, *G. macrocladum*, *L. spinose*, *M. cirrosus*, *P. manginii*, *P. madriti*, *R. similis*, *T. purpureogenus* and *T. trachyspermus* were first found to have pathogenicity for the whitefly.

On the other hand, the pathogenicity of the fungal isolates against *S. litura* seems lower. There were 19 isolates that led to <10% corrected mortality, although the isolates IfGZ4206 of *I. fumosorosea* and IjSC62A03 of *I. javanica* caused mortality of *S. litura* by 78.95% and 63.16%, respectively.

## 4. Discussion

In this research, 490 fungal isolates in 45 species of 24 genera were isolated and identified, which is much more than 213 isolates of 19 species in 12 genera found in the previous report on the South China area [42]. It implies that Southwest China has better EF biodiversity. It is perhaps because the Southwest China has better biodiversity and less interference by humans. As is well known, the area of Yunnan, Guizhou, and Sichuan ranges from northern latitude 21–34° with several mountains over 6000 m in height and climates ranging from tropical to cold zone. Furthermore, the remote Southwest China belongs to the economically undeveloped regions. However, South China locates in tropical to south tropical zone with higher temperature and moisture, the relatively simple climatic conditions make its biodiversity poorer to that of Southwest area. Also, the developed economy and frequent human activity inevitably influence the environment.

The species and isolates found in this study are also more than several reports have reported, which main species are limited to the common EF species, including *Metarhizium* spp., *Beauveria* spp. and *Lacanicillium* spp. isolated by the bait method [43,44]. It may be related to the different experimental methods. The bait methods might be beneficial for selecting the higher fungal pathogens while the trap methods might be more precise for obtaining EF, but the opportunistic EF cannot be selected because they cannot win the competition with the common EF. Obviously, the selective medium used in this research is a reliable method for investigation of EF biodiversity. This method not only finds the EF but also provides the strains as a resource for biocontrol agents.

The results showed that the soil environment has a close influence on EF distribution. Compared to grassy, forest and crop soils, orchard and fallow soils had poorer EF diversity, which is similar to the results of previous studies [42]. This may be due to more human activities in orchards and fallow lands, and the use of chemical pesticides [45,46]. However, the crop soils with frequent human’s interference have better EF diversity than the orchard and fallow soils have. The first reason is mainly related to that cropland has more pests proving more hosts for EF. The second is maybe because these soil samples were collected from the sites covered with more plant species, including various vegetables and grains, etc., but orchard soils are covered with fewer plant species, including mainly mango, orange, Zanthoxylum, pears and peaches, etc.

The SWI data also indicated that Sichuan has better EF biodiversity than that of Yunnan and Guizhou. It might be related to that Sichuan has larger croplands and grasslands, which possess much more EF distribution. Furthermore, the more soil samples collected from Sichuan lead to a larger SWI as well. However, it needs further investigation to determine if different cropping systems, environmental protection, and other factors influence the EF biodiversity in these three regions.

In this study, *P. lilacinum* is the most abundant species with 82 isolates, which is the same as previous research. *Purpureocillium* is a new genus constructed from *Paecilomyces lilacinus* based on its medical significance. *P. lilacinum* is the typical species of this genus, which is considered as a species complex with large intraspecific genetic diversity. This species is not only used extensively to control root-knot nematodes in different crops in China (http://www.chinapesticide.org.cn/hysj/index.jhtml accessed on 7 April 2021), but also is an opportunistic pathogen to infect humans causing keratitis and skin diseases, etc. [47,48]. The prevalence of *P. lilacinum* in soil is probably due to its intraspecific genetic diversity with stronger adaptability to the environment and the large scale application on farms [49]. However, the homologous species *P. lavendulum* with 30 isolates seems much more scare, it is perhaps related to the fact that it cannot tolerate temperature >35 °C [50].

As for *B. bassiana*, *I. fumosorosea*, *I. javanica*, *M. Anisopliae*, *M. carneum* and *M. marquandii* are common EF, which are often found in infected natural insects and are usually used as biological control agents. It is maybe the reason that these EF can be easily isolated from soil [51,52,53]. *Aspergillus* spp. and *Penicillium* spp. are distributed extensively and habitat to live in soil [54]. Many species of them have been reported as EF [20,21,30,32,33].

Furthermore, EF are important medicinal resources. For example, *Ophiocordyceps sinensis*, *I. cicadae* and *I. tenuipes* are expensive traditional Chinese medicines used in East Asia regions [55]. On the other hand, EF provide rich secondary metabolites as potential medicines and pesticides [56,57]. In addition, some EF species are used in the enzymes industry [58].

The bioassay results showed that the 13 species, *A. hispanica*, *A. subramanianii*, *A. tabacinus*, *C. microsporum*, *C. halotolerans*, *G. macrocladum*, *L. spinosa*, *M. cirrosus*, *P. manginii*, *P. madriti*, *R. similis*, *T. purpureogenus* and *T. trachyspermus*, were the first to discover the pathogenicity to the 2nd instar larvae of *B. tabaci* or *S. litura*. However, they only caused a lower corrected mortality range of 4–21%. It may be because they are opportunistic EF. If they infect other pests or have an effect on these pests in the fields that need more research to validate.

## 5. Conclusions

In conclusion, 490 isolates in 45 species of 24 genera were found in 212 soil samples from Guizhou, Sichuan, and Yunnan in Southwest China. Among of them, 32 species (459 isolates) had been reported as EF, while the other 13 species were first found to have pathogenicity to *B. tabaci* and *S. litura*. The dominant EF species were *P. lilacinum*, *M. anisopliae*, *M. marquandii* and *P. citrinum*. Furthermore, the grassy soil has the best EF biodiversity with a Shannon Wiener Index (SWI) of 3.05. The following are the soils from crop, forest, fallow, and orchard with SWIs of 2.88, 2.74, 2.47, and 2.39, respectively. This research will give a new insight for understanding of EF distribution characteristics and their biodiversity conservation and application.

## Figures and Tables

**Table 1:** Information of fungal species isolated.

Species	Isolates	If Reported EF
*Aspergillus flavus*	1	Yes. *Aphis fabae* [19]
*Aspergillus fumigatus*	1	Yes. *Galleria mellonella* [20]
*Arthropsis hispanica*	2	No
*Aspergillus subramanianii*	1	No
*Aspergillus tabacinus*	2	No
*Aspergillus terreus*	23	Yes. *Thaumetopoea pityocampa* [21]
*Beauveria bassiana*	12	Yes
*Brunneochlamydosporium nepalense*	2	Yes. *Myzus persicae* [22]
*Cephalotrichum microsporum*	2	No
*Cladosporium cladosporioides*	9	Yes. *Chrysomya megacephala* [23]
*Cladosporium halotolerans*	2	No
*Cladosporium sphaerospermum*	1	Yes. *Pristiphora abietina* [24]
*Clonostachys pityrodes*	1	Yes. *Caenothabditis elegans* [25]
*Clonostachys rosea*	15	Yes. *Trogoderma granarium* [26]
*Gonytrichum macrocladum*	4	No
*Gliomastix roseogrisea*	1	Yes. *Spodoptera litura*
*Isaria fumosorosea*	3	Yes
*Isaria javanica*	19	Yes
*Lasionectriopsis spinosa*	1	No
*Lecanicillium coprophilum*	10	Yes. *Diaphorina citri* [27]
*Metapochonia bulbillosa*	1	Yes. *Cabera pusaria* [28]
*Metarhizium aciculare*	7	Yes. *Nephotettix virescens* [29]
*Metarhizium anisopliae*	58	Yes
*Metarhizium carneum*	3	Yes
*Metarhizium flavoviride*	5	Yes
*Metarhizium marquandii*	44	Yes
*Microascus cirrosus*	1	No
*Penicillium brevicompactum*	2	Yes. *Scolytus ratzeburgi* [30]
*Penicillium chrysogenum*	1	Yes. *Chaetoptelius vestitus* [31]
*Penicillium citrinum*	37	Yes. *Spodoptera frugiperda* [32]
*Penicillium janthinellum*	11	Yes. *Aedes aegypti* [33]
*Penicillium manginii*	2	No
*Penicillium madriti*	4	No
*Penicillium raperi*	11	Yes. Stigmaeidae mites [34]
*Pochonia chlamydosporia*	3	Yes. *Spartocera dentiventris* [35]
*Pseudogymnoascus pannorum*	30	Yes. *Galleria mellonella* [36]
*Purpureocillium lavendulum*	30	Yes. *Galleria mellonella* [37]
*Purpureocillium lilacinum*	82	Yes. *Anastrepha ludens* [38]
*Rhinocladiella similis*	7	No
*Rhizopus oryzae*	2	Yes. *Leucinodes orbonalis* [39]
*Sarocladium kiliense*	6	Yes. Vine mealybug [40]
*Simplicillium subtropicum*	17	Yes. Hepiaua [41]
*Talaromyces purpureogenus*	1	No
*Talaromyces pinophilum*	11	Yes. *Chaetoptelius vestitus* [31]
*Talaromyces trachyspermus*	2	No

**Table 2:** The fungi isolation and biodiversity of different regions.

Region	Sample Number			Isolation Rate (%)		Isolate Number		EF Species	Shannon Wiener Index (SWI)
	Total	Fungi	EF	Fungi	EF	Total	EF		
Sichuan	124	120	120	96.77	96.77	409	379	29	2.98
Yunnan	40	27	27	67.50	67.50	36	35	10	1.89
Guizhou	48	35	34	72.92	70.83	52	45	11	2.14
Total	212	182	181	85.85	85.38	497	459	32	-

**Table 3:** The fungi isolation and biodiversity of different samples.

Sample Environment	Sample Numbers			Isolation Rate (%)		Isolate Number		EF Species	Shannon Wiener Index
	Total	Fungi	EF	Fungi	EF	Total	EF		
Crop	63	55	54	87.30	85.71	162	151	24	2.88
Forest	41	30	30	73.17	73.17	71	67	18	2.74
Grassy	66	58	58	87.88	87.88	173	155	24	3.05
Orchard	20	19	19	95.00	95.00	44	42	16	2.39
Fallow	22	20	20	90.91	90.91	47	44	19	2.47
Total	212	182	181	85.85	85.38	497	459	32	-

**Table 4:** The pathogenicity of fungal isolates against *B. tabaci* and *S. litura*.

Isolates	Species	Corrected Mortality (%) (7 d Post-Treatment)	
		*B. tabaci*	*S. litura*
AnSC46A07	*Acremonium nepalense*	11.86 ± 1.20 fg	10.53 ± 2.89 e
AhSC46B05	*Arthropsis hispanica*	15.56 ± 2.65 e	1.76 ± 1.67 h
AfSC47B07	*Aspergillus flavus*	12.22 ± 0.58 fg	7.02 ± 1.67 ef
AfSC57C01	*Aspergillus fumigatus*	17.78 ± 3.22 de	10.53 ± 2.89 e
AsSC14C03	*Aspergillus subramanianii*	19.26 ± 0.88 d	1.76 ± 3.33 h
AtSC51B01	*Aspergillus tabacinus*	10.37 ± 0.88 gh	−1.76 ± 1.67 i
BbSC09A01	*Beauveria bassiana*	37.78 ± 2.31 b	36.31 ± 0.00 c
CmSC51B07	*Cephalotrichum microsporum*	10.37 ± 2.19 gh	15.79 ± 2.89 d
ChSC55C04	*Cladosporium halotolerans*	7.04 ± 2.73 h	36.84 ± 2.89 c
CrSC40B04	*Clonostachys rossmaniae*	−4.07 ± 0.33 k	8.77 ± 3.33 ef
GmSC57A06	*Gonytrichum macrocladum*	10.74 ± 4.91 gh	−1.76 ± 3.33 i
IjSC62A03	*Isaria javanica*	57.78 ± 6.93 a	63.16 ± 5.78 b
IfGZ4206	*Isaria fumosorosea*	29.63 ± 4.41 c	78.95 ± 5.00 a
LsSC11A01	*Lasionectriopsis spinosa*	4.44 ± 2.00 i	1.76 ± 1.67 h
LcSC08A01	*Lecanicillium coprophilum*	17.41 ± 0.33 de	5.26 ± 2.89 f
McSC50A01	*Microascus cirrosus*	20.37 ± 4.98 d	5.26 ± 0.00 f
PmSC55C05	*Penicillium madriti*	12.96 ± 1.67 fg	5.26 ± 2.89 f
PmSC62C04	*Penicillium manginii*	8.52 ± 1.20 h	8.77 ± 1.67 ef
PrSC58B03	*Penicillium raperi*	14.44 ± 1.53 ef	5.26 ± 2.89 f
PbSC53C07	*Penicillium brevicompactum*	11.85 ± 1.76 d	5.26 ± 2.89 f
PcSC62C02	*Pochonia chlamydosporia*	10.74 ± 0.33 gh	7.02 ± 1.67 ef
PpSC42A03	*Pseudogymnoascus pannorum*	17.41 ± 0.33 de	−1.76 ± 1.67 i
PlSC29A01	*Purpureocillium lilacinum*	28.15 ± 2.91 c	7.02 ± 4.41 ef
RsSC61B02	*Rhinocladiella similis*	12.96 ± 0.33 fg	5.26 ± 1.67 f
SkSC33A03	*Sarocladium kiliense*	6.67 ± 20.8 h	7.02 ± 1.67 ef
SsSC54A04	*Simplicillium subtropicum*	16.30 ± 3.28 e	10.53 ± 2.89 e
TpSC42B04	*Talaromyces pinophilum*	17.04 ± 6.68 de	10.53 ± 5.78 e
TpSC57A05	*Talaromyces purpureogenus*	13.33 ± 3.01 fg	3.51 ± 1.67 fg
TtSC34B04	*Talaromyces trachyspermus*	20.00 ± 3.08 d	10.53 ± 2.89 e
CK	*-*	1.67 ± 1.67 j	3.33 ± 1.67 fg

The mortality data were mean ± SE, the different letters behind indicate the significant difference (*p* < 0.05) by DMRT. The numbers of 2nd nymph of *B. tabaci* for test were >100 in each treatment, and the numbers of 2nd larvae of *S. litura* for test were 60 in each treatment. The experiment was repeated three times.

## Data Availability

Not applicable.

## Supplementary Materials

The following are available online at https://www.mdpi.com/article/10.3390/jof7110984/s1, Table S1: Information of soil samples collection and fungal isolation.
