# Supplementary material for: Entomopathogenic Fungi Biodiversity in the Soil of Three Provinces Located in Southwest China and First Approach to Evaluate Their Biocontrol Potential
Source: J Fungi (Basel). 2021 Nov 18;7(11):984. doi: 10.3390/jof7110984 (PMC8618793; doi:10.3390/jof7110984)
Supplement: Supplementary file 1 [file jof-07-00984-s001.zip › jof-1444787-supplementary.pdf]

Journal of Fungi

MDPI

Table S1. Information of soil samples collection and fungal isolation.

Sample NO.	Sample Location	Longitude and Latitude	Soil Vegetation	Isolate NO	Species	Genebank Access NO.
SC01	Wenchuan, Sichuan	E103.56,N31.46	crop	PlSC01A01	*Purpureocillium lilacinum*	1. MW113258
				BbSC01A02	*Beauveria bassiana*	2. MW113259
			Grassy	PlSC01B01	*Purpureocillium lilacinum*	3. MW113260
				PpSC01B02	*Pseudogymnoascus pannorum*	4. MW113261
				McSC01B03	*Metarhizium flavoviride*	5. MW113262
SC02	Maoxian, Sichuan	E103.85,N31.73	Grassy	PlSC02A01	*Purpureocillium lilacinum*	6. MW113263
				PpSC02A02	*Pseudogymnoascus pannorum*	7. MW113264
				MaSC02A03	*Pseudogymnoascus pannorum*	8. MW113265
			orchard	MmSC02B01	*Metarhizium marquandii*	9. MW113266
				MaSC02B02	*Metarhizium anisopliae*	10. MW113267
				PpSC02B03	*Metarhizium anisopliae*	11. MW113268
SC03	Aba, Sichuan	E103.73,N32.16	Grassy	MaSC03A02	*Metarhizium anisopliae*	12. MW113269
			orchard	PcSC03B01	*Penicillium citrinum*	13. MW113270
SC04	Aba, Sichuan	E103.52,N32.90	Grassy			14.
SC05	Aba, Sichuan	E103.35,N32.96	Grassy	CcSC05A01	*Cladosporium cladosporioides*	15. MW113271
				PcSC05A03	*Penicillium citrinum*	16. MW113272
SC06	Ruoergai, Sichuan	E102.94,N33.85	Grassy	PpSC06A01	*Pseudogymnoascus pannorum*	17. MW113273
				PlSC06A02	*Purpureocillium lavendulum*	18. MW113274
				PpSC06B02	*Pseudogymnoascus pannorum*	19. MW113275
				MaSC06B03	*Metarhizium anisopliae*	20. MW113276
SC07	Ruoergai, Sichuan	E102.75,N34.00	Grassy	MaSC07A01	*Metarhizium anisopliae*	21. MW113277
				PpSC07A02	*Pseudogymnoascus pannorum*	22. MW113278
SC08	Ruoergai, Sichuan	E102.47,N33.53	Grassy	LcSC08A01	*Lecanicillium coprophilum*	23. MW113279
				PpSC08A02	*Clonostachys rosea*	24. MW113280
SC09	Hongyuan, Sichuan	E102.47,N32.79	Grassy	BbSC09A01	*Beauveria bassiana*	25. MW113281
				MaSC09A02	*Metarhizium anisopliae*	26. MW113282

Sample NO.	Sample Location	Longitude and Latitude	Soil Vegetation	Isolate NO	Species	Genebank Access NO.
				PpSC09A03	*Pseudogymnoascus pannorum*	27. MW113283
SC10	Barkam, Sichuan	E102.11,N31.92	crop	PpSC10A01	*Pseudogymnoascus pannorum*	28. MW113284
SC11	Barkam, Sichuan	E102.04,N31.91	forest	AsSC11A01	*Lasionectriopsis spinosa*	29. MW113285
				PpSC11A02	*Pseudogymnoascus pannorum*	30. MW113286
				PcSC11A03	*Penicillium citrinum*	31. MW113287
				PlSC11A04	*Purpureocillium lilacinum*	32. MW113288
SC12	Barkam, Sichuan	E101.84,N31.84	Grassy	BbSC12A01	*Beauveria bassiana*	33. MW113289
				PpSC12A02	*Pseudogymnoascus pannorum*	34. MW113290
				CrSC12A03	*Clonostachys rosea*	35. MW113291
				CsSC12A04	*Cladosporium sphaerospermum*	36. MW113292
SC13	Rangtang, Sichuan	E101.01,N31.95	Grassy	SC13A02	*Penicillium madriti*	37. MW113293
				MmSC13A05	*Metarhizium marquandii*	38. MW113294
SC14	Luhuo, Sichuan	E100.71,N31.49	Grassy			39.
			crop			40.
			Arable	PcSC14C01	*Penicillium citrinum*	41. MW113295
				PlSC14C02	*Purpureocillium lilacinum*	42. MW113296
				AsSC14C03	*Aspergillus subramanianii*	43. MW113297
SC15	Luhuo, Sichuan	E100.70,N31.38	orchard	PlSC15A01	*Purpureocillium lilacinum*	44. MW113298
				CrSC15A02	*Clonostachys rosea*	45. MW113299
				PpSC15A03	*Pseudogymnoascus pannorum*	46. MW113300
				MmSC15A04	*Metarhizium marquandii*	47. MW113301
				IfSC15A05	*Isaria javanica*	48. MW113302
			Grassy	MaSC15B01	*Metarhizium anisopliae*	49. MW113303
				PlSC15B02	*Purpureocillium lavendulum*	50. MW113304
			crop	MaSC15C01	*Metarhizium anisopliae*	51. MW113305
				PpSC15C02	*Pseudogymnoascus pannorum*	52. MW113306
				PlSC15C03	*Purpureocillium lilacinum*	53. MW113307
SC16	Daofu, Sichuan	E101.20,N30.92	crop	PlSC16A01	*Purpureocillium lilacinum*	54. MW113308
				MaSC16A02	*Metarhizium anisopliae*	55. MW113309

Sample NO.	Sample Location	Longitude and Latitude	Soil Vegetation	Isolate NO	Species	Genebank Access NO.
SC17	Danba, Sichuan	E101.62,N30.55	crop	McSC17A01	*Metarhizium carneum*	56. MW113310
			Grassy	PpSC17A02	*Pseudogymnoascus pannorum*	57. MW113311
				MaSC17A05	*Metarhizium anisopliae*	58. MW113312
SC18	Danba, Sichuan	E101.72,N30.60	crop	PlSC18A01	*Purpureocillium lilacinum*	59. MW113313
				PpSC18A03	*Pseudogymnoascus pannorum*	60. MW113314
				MmSC18A04	*Metarhizium marquandii*	61. MW113315
				MaSC18A05	*Metarhizium anisopliae*	62. MW113316
			Grassy	PpSC18B01	*Pseudogymnoascus pannorum*	63. MW113317
				BbSC18B02	*Beauveria bassiana*	64. MW113318
				McSC18B03	*Metarhizium carneum*	65. MW113319
SC19	Danba, Sichuan	E101.97,N30.94	crop	MaSC19A01	*Metarhizium anisopliae*	66. MW113320
				MmSC19A02	*Metarhizium marquandii*	67. MW113321
			Grassy	MmSC19B01	*Metarhizium marquandii*	68. MW113322
				MaSC19B03	*Metarhizium anisopliae*	69. MW113323
				CcSC19B04	*Cladosporium cladosporioides*	70. MW113324
				PcSC19B05	*Penicillium citrinum*	71. MW113325
				SkSC19B07	*Sarocladium kiliense*	72. MW113326
SC20	Xiaojin, Sichuan	E102.66,N30.97	crop	PcSC20A02	*Penicillium citrinum*	73. MW113327
				BbSC20A03	*Beauveria bassiana*	74. MW113328
				PpSC20A05	*Pseudogymnoascus pannorum*	75. MW113329
				SkSC20A06	*Sarocladium kiliense*	76. MW113330
				MmSC20A07	*Metarhizium marquandii*	77. MW113331
				PlSC20A08	*Purpureocillium lavendulum*	78. MW113332
						79.
				PpSC20B02	*Pseudogymnoascus pannorum*	80. MW113333
				MmSC20B04	*Metarhizium marquandii*	81. MW113334
SC21	Aba, Sichuan	E102.52,N31.15	forest	PlSC21A01	*Purpureocillium lilacinum*	82. MW113335
				PcSC21A02	*Penicillium citrinum*	83. MW113336
SC22	Xiaojin, Sichuan	E102.41,N30.51	Grassy	MaSC22A01	*Metarhizium anisopliae*	84. MW113337

Sample NO.	Sample Location	Longitude and Latitude	Soil Vegetation	Isolate NO	Species	Genebank Access NO.
				SsSC22A02	*Simplicillium subtropicum*	85. MW113338
				PpSC22A03	*Pseudogymnoascus pannorum*	86. MW113339
SC23	Ya'an, Sichuan	E102.73,N30.77	Grassy	BbSC23A01	*Beauveria bassiana*	87. MW113340
				PpSC23A02	*Pseudogymnoascus pannorum*	88. MW113341
				MaSC23A03	*Metarhizium anisopliae*	89. MW113342
			crop	PpSC24A01	*Pseudogymnoascus pannorum*	90. MW113343
SC24	Lushan, Sichuan	E102.91,N30.11	Grassy	PmSC24A02	*Penicillium manginii*	91. MW113344
				MaSC24A03	*Metarhizium anisopliae*	92. MW113345
				MmSC24A04	*Metarhizium marquandii*	93. MW113346
			crop			94.
SC25	Tianquan, Sichuan	E102.79,N30.02	Arable	PcSC25A01	*Pochonia chlamydosporia*	95. MW113347
				PpSC25A02	*Pseudogymnoascus pannorum*	96. MW113348
				PcSC25A03	*Penicillium citrinum*	97. MW113349
				MaSC25A04	*Metarhizium anisopliae*	98. MW113350
				PlSC25A05	*Purpureocillium lilacinum*	99. MW113351
SC26	Luding, Sichuan	E102.23,N29.96	Grassy	PlSC26A02	*Purpureocillium lilacinum*	100. MW113352
				MaSC26A03	*Metarhizium anisopliae*	101. MW113353
SC27	Kangding, Sichuan	E101.95,N30.00	forest	MmSC27A02	*Metarhizium marquandii*	102. MW113354
				MaSC27A03	*Metarhizium anisopliae*	103. MW113355
				PpSC27A04	*Pseudogymnoascus pannorum*	104. MW113356
				PcSC27A06	*Metapochonia bulbillosa*	105. MW113357
				SsSC27A07	*Simplicillium subtropicum*	106. MW113358
			Grassy	SsSC27B01	*Simplicillium subtropicum*	107. MW113359
				PpSC27B03	*Pseudogymnoascus pannorum*	108. MW113360
				PcSC27B04	*Penicillium citrinum*	109. MW113361
				McSC27B05	*Metarhizium carneum*	110. MW113362
				MaSC27B07	*Metarhizium anisopliae*	111. MW113363
			crop	PlSC27C01	*Purpureocillium lilacinum*	112. MW113364
				SC27C03	*Penicillium raperi*	113. MW113365

Sample NO.	Sample Location	Longitude and Latitude	Soil Vegetation	Isolate NO	Species	Genebank Access NO.
				CrSC27C04	*Clonostachys rosea*	114. MW113366
				SsSC27C05	*Simplicillium subtropicum*	115. MW113367
				PcSC27C07	*Penicillium citrinum*	116. MW113368
				PpSC27C08	*Pseudogymnoascus pannorum*	117. MW113369
				SC27D02	*Penicillium raperi*	118. MW113370
			Grassy	PlSC27D03	*Purpureocillium lilacinum*	119. MW113371
SC28	Kangding, Sichuan	E101.63,N30.08	Arable	PpSC28A01	*Pseudogymnoascus pannorum*	120. MW113372
				PlSC28A02	*Purpureocillium lilacinum*	121. MW113373
				PcSC28A03	*Penicillium citrinum*	122. MW113374
				AtSC28A04	*Aspergillus terreus*	123. MW113375
				MaSC28A05	*Metarhizium anisopliae*	124. MW113376
			Grassy	PcSC28B01	*Penicillium citrinum*	125. MW113377
				PlSC28B02	*Purpureocillium lavendulum*	126. MW113378
				SsSC28B03	*Simplicillium subtropicum*	127. MW113379
				CrSC28B04	*Clonostachys rosea*	128. MW113380
SC29	Yajiang, Sichuan	E100.66,N30.15	Grassy	PlSC29A01	*Purpureocillium lilacinum*	129. MW113381
SC30	Litang, Sichuan	E100.24,N29.99	Grassy	PcSC30A02	*Penicillium citrinum*	130. MW113382
				PlSC30A05	*Purpureocillium lilacinum*	131. MW113383
SC31	Litang, Sichuan	E099.88,N30.22	Grassy	PlSC31A02	*Purpureocillium lilacinum*	132. MW113384
				PjSC31A03	*Penicillium janthinellum*	133. MW113385
SC32	Batang, Sichuan	E099.41,N30.27	crop	PlSC32A01	*Purpureocillium lavendulum*	134. MW113386
				SsSC32A02	*Simplicillium subtropicum*	135. MW113387
				MaSC32B01	*Metarhizium anisopliae*	136. MW113388
				IfSC32B02	*Isaria javanica*	137. MW113389
			Grassy	PcSC32C01	*Penicillium citrinum*	138. MW113390
				IfSC32C03	*Isaria javanica*	139. MW113391
SC33	Batang, Sichuan	E099.01,N29.27	Grassy	PlSC33A01	*Purpureocillium lilacinum*	140. MW113392
				AtSC33A02	*Aspergillus terreus*	141. MW113393

Sample NO.	Sample Location	Longitude and Latitude	Soil Vegetation	Isolate NO	Species	Genebank Access NO.
				SkSC33A03	*Sarocladium kiliense*	142. MW113394
				PlSC33B01	*Purpureocillium lilacinum*	143. MW113395
				MaSC33B02	*Purpureocillium lavendulum*	144. MW113396
				CrSC33B03	*Clonostachys rosea*	145. MW113397
				MmSC33B04	*Metarhizium marquandii*	146. MW113398
				PcSC33B05	*Penicillium citrinum*	147. MW113399
				SsSC33B06	*Simplicillium subtropicum*	148. MW113400
				BbSC33B07	*Beauveria bassiana*	149. MW113401
SC34	Ya'an, Sichuan	E102.89,N29.81	forest	IfSC34A01	*Isaria javanica*	150. MW113402
				MaSC34A02	*Metarhizium anisopliae*	151. MW113403
			crop	MaSC34B02	*Metarhizium anisopliae*	152. MW113404
				MmSC34B03	*Metarhizium marquandii*	153. MW113405
				TtSC34B04	*Talaromyces trachyspermus*	154. MW113406
SC35	Longchang, Sichuan	E105.31,N29.32	Grassy	IjSC35A03	*Isaria javanica*	155. MW113407
				MmSC35A04	*Cladosporium cladosporioides*	156. MW113408
			crop	PlSC35B01	*Purpureocillium lavendulum*	157. MW113409
				SC35B02	*Penicillium raperi*	158. MW113410
				IfSC35B04	*Isaria javanica*	159. MW113411
SC36	Hejiang, Sichuan	E105.88,N28.90	Grassy	CcSC36A01	*Cladosporium cladosporioides*	160. MW113412
				MaSC36A02	*Metarhizium anisopliae*	161. MW113413
				AtSC36A04	*Aspergillus terreus*	162. MW113414
				SC36B02	*Penicillium raperi*	163. MW113415
			crop	PlSC36B01	*Purpureocillium lilacinum*	164. MW113416
			orchard	PlSC36C01	*Purpureocillium lilacinum*	165. MW113417
				PpSC36C04	*Purpureocillium lilacinum*	166. MW113418
				MaSC36C06	*Metarhizium anisopliae*	167. MW113419
				PsSC36C07	*Penicillium janthinellum*	168. MW113420
SC37	Changning, Sichuan	E105.02,N28.73	crop	PlSC37A03	*Purpureocillium lilacinum*	169. MW113421

Sample NO.	Sample Location	Longitude and Latitude	Soil Vegetation	Isolate NO	Species	Genebank Access NO.
				PcSC37A04	*Penicillium janthinellum*	170. MW113422
				AtSC37A05	*Aspergillus terreus*	171. MW113423
				MmSC37A06	*Metarhizium marquandii*	172. MW113424
				SC37A08	*Penicillium raperi*	173. MW113425
			forest	PcSC37B01	*Penicillium citrinum*	174. MW113426
				PjC37B02	*Penicillium janthinellum*	175. MW113427
				SC37B04	*Penicillium raperi*	176. MW113428
			Grassy	PjSC37C01	*Penicillium janthinellum*	177. MW113429
				PlSC37C02	*Purpureocillium lilacinum*	178. MW113430
SC38	Yibin, Sichuan	E104.93,N28.83	forest	MmSC38A02	*Metarhizium marquandii*	179. MW113431
				PlSC38A03	*Purpureocillium lavendulum*	180. MW113432
			orchard	PlSC38B01	*Purpureocillium lilacinum*	181. MW113433
				MaSC38B03	*Metarhizium anisopliae*	182. MW113434
			Grassy	PcSC38C01	*Penicillium citrinum*	183. MW113435
				MaSC38C02	*Metarhizium anisopliae*	184. MW113436
				PlSC38C03	*Purpureocillium lavendulum*	185. MW113437
				PhSC38C04	*Gonytrichum macrocladum*	186. MW113438
				MmSC38C05	*Metarhizium marquandii*	187. MW113439
SC39	Xuzhou, Sichuan	E104.38,N28.93	crop	PlSC39A01	*Purpureocillium lilacinum*	188. MW113440
				PcSC39A02	*Penicillium citrinum*	189. MW113441
				CrSC39A04	*Clonostachys rosea*	190. MW113442
			orchard	MaSC39B01	*Metarhizium anisopliae*	191. MW113443
				PcSC39B02	*Penicillium citrinum*	192. MW113444
SC40	Qianwei, Sichuan	E103.95,N29.17	crop	PlSC40A01	*Purpureocillium lilacinum*	193. MW113445
				MmSC40A02	*Metarhizium marquandii*	194. MW113446
				PcSC40A03	*Penicillium citrinum*	195. MW113447
			Grassy	MmSC40B01	*Metarhizium marquandii*	196. MW113448
				RsSC40B02	*Rhinocladiella similis*	197. MW113449
				PlSC40B03	*Purpureocillium lilacinum*	198. MW113450

Sample NO.	Sample Location	Longitude and Latitude	Soil Vegetation	Isolate NO	Species	Genebank Access NO.
				SC40B06	*Acremonium nepalense*	199. MW113451
SC41	Jingyan, Sichuan	E104.01,N29.53	Arable	MmSC41A01	*Metarhizium marquandii*	200. MW113452
				PlSC41A02	*Purpureocillium lavendulum*	201. MW113453
				RsSC41A03	*Rhinocladiella similis*	202. MW113454
			crop	PlSC41B02	*Purpureocillium lilacinum*	203. MW113455
			forest	MmSC41C01	*Metarhizium marquandii*	204. MW113456
				PcSC41C02	*Penicillium citrinum*	205. MW113457
				PlSC41C03	*Purpureocillium lilacinum*	206. MW113458
SC42	Rongxian, Sichuan	E104.36,N29.44	crop	MmSC42A01	*Metarhizium marquandii*	207. MW113459
				MaSC42A02	*Metarhizium anisopliae*	208. MW113460
				PpSC42A03	*Pseudogymnoascus pannorum*	209. MW113461
			Grassy	RsSC42B02	*Rhinocladiella similis*	210. MW113462
				PlSC42B03	*Purpureocillium lilacinum*	211. MW113463
				PpSC42B04	*Talaromyces pinophilum*	212. MW113464
			crop	RsSC42C01	*Rhinocladiella similis*	213. MW113465
				PlSC42C02	*Purpureocillium lilacinum*	214. MW113466
				MmSC42C04	*Metarhizium marquandii*	215. MW113467
				PcSC42C05	*Penicillium citrinum*	216. MW113468
				SsSC42C06	*Simplicillium subtropicum*	217. MW113469
				MaSC42C07	*Metarhizium anisopliae*	218. MW113470
				PhSC42C08	*Gonytrichum macrocladum*	219. MW113471
SC43	Weiyuan, Sichuan	E104.62,N29.52	forest	MmSC43A01	*Metarhizium marquandii*	220. MW113472
				AtSC43A02	*Aspergillus terreus*	221. MW113473
				MaSC43A03	*Metarhizium anisopliae*	222. MW113474
				PlSC43A04	*Purpureocillium lilacinum*	223. MW113475
				CcSC43A05	*Cladosporium cladosporioides*	224. MW113476
				PcSC43A06	*Penicillium citrinum*	225. MW113477
			Arable	SC43B01	*Penicillium raperi*	226. MW113478

Sample NO.	Sample Location	Longitude and Latitude	Soil Vegetation	Isolate NO	Species	Genebank Access NO.
			crop	PcSC43C01	*Penicillium citrinum*	227. MW113479
				MaSC43C03	*Metarhizium anisopliae*	228. MW113480
SC44	Renshou, Sichuan	E104.18,N29.96	Grassy	PlSC44A01	*Purpureocillium lilacinum*	229. MW113481
				AtSC44A02	*Aspergillus terreus*	230. MW113482
				MaSC44A03	*Metarhizium anisopliae*	231. MW113483
				PcSC44A04	*Penicillium citrinum*	232. MW113484
			crop	MaSC44B01	*Metarhizium anisopliae*	233. MW113485
				TpSC44B02	*Talaromyces pinophilum*	234. MW113486
				PlSC44B04	*Purpureocillium lilacinum*	235. MW113487
				PhSC44B05	*Gonytrichum macrocladum*	236. MW113488
				SkSC44B08	*Sarocladium kiliense*	237. MW113489
				CrSC44B09	*Clonostachys rosea*	238. MW113490
				AtSC44B10	*Aspergillus terreus*	239. MW113491
				IjSC44B11	*Isaria javanica*	240. MW113492
SC45	Shuangliu, Chendu	E104.12,N30.30	forest	CcSC45A01	*Cladosporium cladosporioides*	241. MW113493
				PcSC45A02	*Penicillium citrinum*	242. MW113494
				PlSC45A03	*Purpureocillium lilacinum*	243. MW113495
				MmSC45A04	*Metarhizium marquandii*	244. MW113496
				SC45A05	*Penicillium madriti*	245. MW113497
				AtSC45A06	*Aspergillus terreus*	246. MW113498
			Grassy	AtSC45B01	*Aspergillus terreus*	247. MW113499
				MaSC45B03	*Metarhizium anisopliae*	248. MW113500
				CcSC45B04	*Cladosporium cladosporioides*	249. MW113501
				PlSC45B05	*Purpureocillium lavendulum*	250. MW113502
			crop	IjSC45C01	*Isaria javanica*	251. MW113503
				PlSC45C03	*Purpureocillium lilacinum*	252. MW113504
				CrSC45C06	*Clonostachys pityrodes*	253. MW113505
SC46	Guanghan, Sichuan	E104.32,N30.99	Grassy	SC46A01	*Aspergillus tabacinus*	254. MW113506

Sample NO.	Sample Location	Longitude and Latitude	Soil Vegetation	Isolate NO	Species	Genebank Access NO.
				SC46A02	*Cephalotrichum microsporum*	255. MW113507
				MaSC46A04	*Metarhizium anisopliae*	256. MW113508
				MmSC46A06	*Metarhizium marquandii*	257. MW113509
				AnSC46A07	*Acremonium nepalense*	258. MW113510
				AtSC46A09	*Aspergillus terreus*	259. MW113511
				PlSC46A10	*Purpureocillium lilacinum*	260. MW113512
			crop	SsSC46B02	*Simplicillium subtropicum*	261. MW113513
				CrSC46B03	*Clonostachys rosea*	262. MW113514
				MaSC46B04	*Metarhizium anisopliae*	263. MW113515
				SC46B05	*Arthropsis hispanica*	264. MW113516
				PlSC46B06	*Purpureocillium lilacinum*	265. MW113517
				MmSC46B07	*Metarhizium marquandii*	266. MW113518
				RoSC46C03	*Rhizopus oryzae*	267. MW113519
				PlSC46C04	*Purpureocillium lilacinum*	268. MW113520
				MmSC46C05	*Metarhizium marquandii*	269. MW113521
SC47	Mianyang, Sichuan	E104.87,N31.56	crop	PjSC47A01	*Penicillium janthinellum*	270. MW113522
				PlSC47A02	*Purpureocillium lilacinum*	271. MW113523
			Grassy	AtSC47B01	*Aspergillus terreus*	272. MW113524
				CrSC47B02	*Clonostachys rosea*	273. MW113525
				PlSC47B03	*Purpureocillium lilacinum*	274. MW113526
				PsSC47B05	*Penicillium janthinellum*	275. MW113527
				PcSC47B06	*Penicillium chrysogenum*	276. MW113528
				AfSC47B07	*Aspergillus flavus*	277. MW113529
SC48	Zitong, Sichuan	E105.20,N31.69	forest	PlSC48A01	*Purpureocillium lilacinum*	278. MW113530
				PcSC48A02	*Penicillium citrinum*	279. MW113531
				IjSC48A03	*Isaria javanica*	280. MW113532
SC49	Jiange, Sichuan	E105.26,N31.84	Grassy	SC49A01	*Penicillium madriti*	281. MW113533
				PlSC49A02	*Purpureocillium lilacinum*	282. MW113534

Sample NO.	Sample Location	Longitude and Latitude	Soil Vegetation	Isolate NO	Species	Genebank Access NO.
				SsSC49A04	*Simplicillium subtropicum*	283. MW113535
			Arable	PcSC49B01	*Penicillium citrinum*	284. MW113536
				RoSC49B03	*Rhizopus oryzae*	285. MW113537
				PlSC49B04	*Purpureocillium lilacinum*	286. MW113538
			crop	PlSC49C02	*Purpureocillium lilacinum*	287. MW113539
				PcSC49C03	*Pochonia chlamydosporia*	288. MW113540
				SkSC49C04	*Sarocladium kiliense*	289. MW113541
SC50	Jiange, Sichuan	E105.48,N32.12	crop	McSC50A01	*Microascus cirrosus*	290. MW113542
				MaSC50A02	*Metarhizium anisopliae*	291. MW113543
				PlSC50A03	*Purpureocillium lilacinum*	292. MW113544
				PcSC50A04	*Penicillium citrinum*	293. MW113545
				PjSC50A05	*Penicillium janthinellum*	294. MW113546
				CrSC50A07	*Clonostachys rosea*	295. MW113547
			Grassy	IjSC50B03	*Isaria javanica*	296. MW113548
			orchard	MmSC50C02	*Purpureocillium lilacinum*	297. MW113549
				LcSC50C03	*Lecanicillium coprophilum*	298. MW113550
				PcSC50C04	*Penicillium citrinum*	299. MW113551
				MaSC50C05	*Metarhizium anisopliae*	300. MW113552
				TpSC50C06	*Talaromyces pinophilum*	301. MW113553
				AtSC50C07	*Aspergillus terreus*	302. MW113554
				BbSC50C08	*Beauveria bassiana*	303. MW113555
SC51	Guangyuan, Sichuan	E106.11,N32.69	forest	TpSC51A01	*Talaromyces pinophilum*	304. MW113556
				MmSC51A02	*Metarhizium marquandii*	305. MW113557
				SsSC51A03	*Simplicillium subtropicum*	306. MW113558
				PlSC51A04	*Purpureocillium lilacinum*	307. MW113559
				SC51A05	*Penicillium raperi*	308. MW113560
				AtSC51B01	*Aspergillus tabacinus*	309. MW113561
			crop	PlSC51B02	*Purpureocillium lavendulum*	310. MW113562

Sample NO.	Sample Location	Longitude and Latitude	Soil Vegetation	Isolate NO	Species	Genebank Access NO.
				IjSC51B04	*Isaria javanica*	311. MW113563
				PjSC51B05	*Penicillium janthinellum*	312. MW113564
				MaSC51B06	*Metarhizium anisopliae*	313. MW113565
				CmSC51B07	*Cephalotrichum microsporum*	314. MW113566
			orchard	SsSC51C01	*Simplicillium subtropicum*	315. MW113567
				PlSC51C02	*Purpureocillium lilacinum*	316. MW113568
				MmSC51C03	*Metarhizium marquandii*	317. MW113569
SC52	Wangcang, Sichuan	E106.40,N32.25	crop	CrSC52A01	*Clonostachys rosea*	318. MW113570
			Arable	PlSC52B01	*Purpureocillium lilacinum*	319. MW113571
			Grassy	MaSC52C01	*Metarhizium anisopliae*	320. MW113572
				AtSC52C02	*Aspergillus terreus*	321. MW113573
				IjSC52C03	*Isaria javanica*	322. MW113574
				PlSC52C04	*Purpureocillium lilacinum*	323. MW113575
				PpSC52C06	*Talaromyces pinophilum*	324. MW113576
SC53	Bazhong, Sichuan	E106.79,N31.86	crop	CrSC53A01	*Clonostachys rosea*	325. MW113577
				IjSC53A04	*Isaria javanica*	326. MW113578
			Grassy	AtSC53B01	*Aspergillus terreus*	327. MW113579
				PcSC53B02	*Penicillium citrinum*	328. MW113580
				IjSC53B05	*Isaria javanica*	329. MW113581
			Arable	IjSC53C02	*Isaria javanica*	330. MW113582
				PlSC53C03	*Purpureocillium lilacinum*	331. MW113583
				PpSC53C06	*Talaromyces pinophilum*	332. MW113584
				PbSC53C07	*Penicillium brevicompactum*	333. MW113585
				AtSC53C08	*Aspergillus terreus*	334. MW113586
SC54	Pingchang, Sichuan	E107.04,N31.68	forest	IfSC54A03	*Isaria javanica*	335. MW113587
				SsSC54A04	*Simplicillium subtropicum*	336. MW113588
				PcSC54A05	*Penicillium citrinum*	337. MW113589
				AtSC54A06	*Aspergillus terreus*	338. MW113590

Sample NO.	Sample Location	Longitude and Latitude	Soil Vegetation	Isolate NO	Species	Genebank Access NO.
			crop	TpSC54B01	*Talaromyces pinophilum*	339. MW113591
				PlSC54B02	*Purpureocillium lilacinum*	340. MW113592
				SsSC54B04	*Simplicillium subtropicum*	341. MW113593
				MmSC54B05	*Metarhizium marquandii*	342. MW113594
				PcSC54B06	*Penicillium citrinum*	343. MW113595
SC55	Dazhou, Sichuan	E107.48,N31.13	crop	SsSC55A01	*Simplicillium subtropicum*	344. MW113596
			Arable	SkSC55B01	*Sarocladium kiliense*	345. MW113597
				PlSC55B02	*Purpureocillium lavendulum*	346. MW113598
				CcSC55B06	*Cladosporium cladosporioides*	347. MW113599
				PpSC55B07	*Talaromyces pinophilum*	348. MW113600
				SC55B08	*Penicillium raperi*	349. MW113601
			Grassy	PlSC55C01	*Purpureocillium lilacinum*	350. MW113602
				MaSC55C02	*Metarhizium anisopliae*	351. MW113603
				ChSC55C04	*Cladosporium halotolerans*	352. MW113604
				SC55C05	*Penicillium madriti*	353. MW113605
				AtSC55C07	*Aspergillus terreus*	354. MW113606
SC56	Dazhou, Sichuan	E107.27,N30.78	Grassy	PjSC56A03	*Penicillium janthinellum*	355. MW113607
			crop	PpSC56B01	*Pseudogymnoascus pannorum*	356. MW113608
				PcSC56B04	*Penicillium janthinellum*	357. MW113609
SC57	Guang'an, Sichuan	E106.72,N30.29	Grassy	PlSC57A01	*Purpureocillium lilacinum*	358. MW113610
				PpSC57A03	*Pseudogymnoascus pannorum*	359. MW113611
				RsSC57A04	*Rhinocladiella similis*	360. MW113612
				TpSC57A05	*Talaromyces purpureogenus*	361. MW113613
				PhSC57A06	*Gonytrichum macrocladum*	362. MW113614
			crop	SC57A08	*Penicillium raperi*	363. MW113615
				MmSC57A09	*Metarhizium marquandii*	364. MW113616
				PcSC57A12	*Penicillium citrinum*	365. MW113617
			Arable	PpSC57B01	*Talaromyces pinophilum*	366. MW113618

Sample NO.	Sample Location	Longitude and Latitude	Soil Vegetation	Isolate NO	Species	Genebank Access NO.
				PlSC57B02	*Purpureocillium lilacinum*	367. MW113619
				AtSC57B03	*Aspergillus terreus*	368. MW113620
			crop	AfSC57C01	*Aspergillus fumigatus*	369. MW113621
				SsSC57C03	*Simplicillium subtropicum*	370. MW113622
SC58	Nanchong, Sichuan	E106.34,N30.79	Grassy	PlSC58A01	*Purpureocillium lilacinum*	371. MW113623
				PcSC58A02	*Penicillium citrinum*	372. MW113624
				SsSC58A03	*Simplicillium subtropicum*	373. MW113625
				AtSC58A04	*Aspergillus terreus*	374. MW113626
			crop	PpSC58B01	*Pseudogymnoascus pannorum*	375. MW113627
				PlSC58B02	*Purpureocillium lilacinum*	376. MW113628
				SC58B03	*Penicillium raperi*	377. MW113629
				AtSC58B07	*Aspergillus terreus*	378. MW113630
SC59	Nanchong, Sichuan	E106.15,N30.64	orchard	PcSC59A01	*Penicillium citrinum*	379. MW113631
				PlSC59A02	*Purpureocillium lilacinum*	380. MW113632
			forest	PlSC59B01	*Purpureocillium lilacinum*	381. MW113633
				AtSC59B02	*Aspergillus terreus*	382. MW113634
SC60	Pengxi, Sichuan	E105.97,N30.43	crop	CcSC60A01	*Cladosporium cladosporioides*	383. MW113635
				PbSC60A03	*Penicillium brevicompactum*	384. MW113636
				CrSC60B01	*Clonostachys rosea*	385. MW113637
				MmSC60B03	*Metarhizium marquandii*	386. MW113638
			Grassy	PpSC60C02	*Talaromyces pinophilum*	387. MW113639
				PlSC60C05	*Purpureocillium lilacinum*	388. MW113640
SC61	Suining, Sichuan	E105.50,N30.44	crop	CrSC61A01	*Clonostachys rosea*	389. MW113641
				PlSC61A02	*Purpureocillium lilacinum*	390. MW113642
				PpSC61B01	*Talaromyces pinophilum*	391. MW113643
				RsSC61B02	*Rhinocladiella similis*	392. MW113644
				PlSC61B03	*Purpureocillium lilacinum*	393. MW113645
				MaSC61B05	*Metarhizium anisopliae*	394. MW113646

Sample NO.	Sample Location	Longitude and Latitude	Soil Vegetation	Isolate NO	Species	Genebank Access NO.
			Grassy	PlSC61C01	*Purpureocillium lilacinum*	395. MW113647
				MmSC61C02	*Metarhizium marquandii*	396. MW113648
SC62	Lezhi, Sichuan	E105.17,N30.32	crop	AtSC62A01	*Aspergillus terreus*	397. MW113649
				IfSC62A03	*Isaria javanica*	398. MW113650
				MmSC62A05	*Metarhizium marquandii*	399. MW113651
				PlSC62A06	*Purpureocillium lilacinum*	400. MW113652
				PlSC62B01	*Purpureocillium lilacinum*	401. MW113653
				MaSC62B04	*Metarhizium anisopliae*	402. MW113654
				AtSC62B05	*Aspergillus terreus*	403. MW113655
			Grassy	PlSC62C01	*Purpureocillium lilacinum*	404. MW113656
				PcSC62C02	*Pochonia chlamydosporia*	405. MW113657
				MmSC62C03	*Metarhizium marquandii*	406. MW113658
				PmSC62C04	*Penicillium manginii*	407. MW113659
				PpSC62C05	*Pseudogymnoascus pannorum*	408. MW113660
SC63	Jianyang, Sichuan	E104.81,N30.30	crop	MmSC63A01	*Metarhizium marquandii*	409. MW113661
				TtSC63A02	*Talaromyces trachyspermus*	410. MW113662
				PlSC63B01	*Purpureocillium lilacinum*	411. MW113663
			Grassy	SC63C01	*Arthropsis hispanica*	412. MW113664
				MmSC63C02	*Metarhizium marquandii*	413. MW113665
				RsSC63C03	*Rhinocladiella similis*	414. MW113666
YN01	Jinghong, Yunnan	E100.89,N22.03	forest	PcYN0101	*Penicillium citrinum*	415. MH483736
				PlYN0102	*Purpureocillium lilacinum*	416. MH483737
			orchard	BbYN0103	*Beauveria bassiana*	417. MH483738
YN02	Jinghong, Yunnan	E101.06,N22.45	forest	BbYN0201	*Beauveria bassiana*	418. MH483739
				P.YN0202	*Metarhizium aciculare*	419. MH483740
			orchard	PlYN0203	*Purpureocillium lilacinum*	420. MH483741
				MaYN0204	*Metarhizium anisopliae*	421. MH483742
YN03	Simao, Yunnan	E101.01,N22.6	crop	PlYN0301	*Purpureocillium lilacinum*	422. MH483743

Sample NO.	Sample Location	Longitude and Latitude	Soil Vegetation	Isolate NO	Species	Genebank Access NO.
YN04	Dali, Yunnan	E100.27,N25.71	crop	PlYN0401	*Purpureocillium lilacinum*	423. MH483744
				PlYN0402	*Purpureocillium lavendulum*	424. MH483745
			Arable	A.YN0403	*Gliomastix roseogrisea*	425. MH483768
YN59	Shizong, Yunnan	E104.24,N24.74	forest	PlYN5901	*Purpureocillium lavendulum*	426. MH483746
YN60	Qiubei, Yunnan	E104.16,N24.12	forest			427.
YN61	Guangnan, Yunnan	E104.79,N23.75	Grassy			428.
YN62	Shizong, Yunnan	E104.33,N24.55	forest			429.
YN63	Guangnan, Yunnan	E105.06,N24.3	orchard			430.
YN64	Yanshan, Yunnan	E104.34,N23.67	forest			431.
YN65	Kaiyuan, Yunnan	E103.28,N23.86	crop			432.
YN66	Gejiu, Yunnan	E103.2,N23.55	forest	PlYN6601	*Purpureocillium lavendulum*	433. MH483747
YN67	Shiping, Yunnan	E102.65,N23.66	Grassy	PlYN6701	*Purpureocillium lavendulum*	434. MH483748
YN68	Yuanjiang, Yunnan	E101.87,N23.66	Arable			435.
YN69	Mojiang, Yunnan	E101.61,N23.41	crop			436.
YN70	Ninger, Yunnan	E101.14,N23.19	crop	PlYN7001	*Purpureocillium lilacinum*	437. MH483749
				IfYN7003	*Isaria javanica*	438. MH483750
YN71	Ninger, Yunnan	E101.03,N23.1	crop			439.
YN72	Jinggu, Yunnan	E100.92,N23.28	forest			440.
YN73	Jinggu, Yunnan	E100.65,N23.5	forest			441.
YN74	Jinggu, Yunnan	E100.47,N23.57	crop	PcYN7401	*Metarhizium aciculare*	442. MH483751
				PlYN7402	*Purpureocillium lilacinum*	443. MH483752
YN75	Shuangjiang, Yunnan	E100.15,N23.52	orchard	PcYN7501	*Metarhizium aciculare*	444. MH483753
YN76	Yunxian, Yunnan	E100.01,N24.18	crop	PlYN7601	*Purpureocillium lavendulum*	445. MH483754
YN77	Yongde, Yunnan	E99.61,N23.94	crop	PcYN7701	*Metarhizium aciculare*	446. MH483755
				PlYN7703	*Purpureocillium lilacinum*	447. MH483756
YN78	Yongde, Yunnan	E99.43,N24.12	forest	PcYN7801	*Metarhizium aciculare*	448. MH483757
YN79	Yongde, Yunnan	E99.34,N24.39	forest	PlYN7901	*Purpureocillium lavendulum*	449. MH483758
YN80	Shidian, Yunnan	E99.19,N24.67	crop	PcYN8001	*Metarhizium aciculare*	450. MH483759
				PlYN8002	*Purpureocillium lilacinum*	451. MH483760
YN81	Longling, Yunnan	E98.87,N24.79	forest	PcYN8101	*Metarhizium aciculare*	452. MH483761

Sample NO.	Sample Location	Longitude and Latitude	Soil Vegetation	Isolate NO	Species	Genebank Access NO.
				PlYN8102	*Purpureocillium lilacinum*	453. MH483762
YN82	Longling, Yunnan	E98.77,N24.83	orchard	PlYN8202	*Purpureocillium lilacinum*	454. MH483763
YN83	Tengchong, Yunnan	E98.58,N24.94	forest			455.
				PmYN8303	*Metarhizium marquandii*	456. MH483764
			Arable	MaYN8304	*Metarhizium anisopliae*	457. MH483765
				MfYN8305	*Metarhizium flavoviride*	458. MH483766
YN84	Longyang, Yunnan	E99.2,N25.31	forest	PlYN8401	*Purpureocillium lavendulum*	459. MH483767
YN85	Yangbi, Yunnan	E99.86,N25.46	crop			460.
YN86	Zunzhuang, Yunnan	E100.32,N25.7	crop			461.
YN87	Eryuan, Yunnan	E99.99,N26.1	crop	BbYN8702	*Beauveria bassiana*	462.
YN88	Xiangyun, Yunnan	E100.88,N25.38	crop	PlYN8801	*Purpureocillium lavendulum*	463. MH483770
YN89	Lufeng, Yunnan	E102.11,N24.94	forest			464.
YN90	Malong, Yunnan	E103.32,N25.37	crop	PlYN9001	*Purpureocillium lavendulum*	465. MH483771
GZ34	Dushan, Guizhou	E107.44,N25.35	Grassy			466.
GZ35	Dushan, Guizhou	E107.57,N25.85	Grassy	PlGZ3501	*Purpureocillium lavendulum*	467. MH483772
				P.GZ3502	*Purpureocillium lavendulum*	468. MH483773
			Arable	MaGZ3503	*Metarhizium anisopliae*	469. MH483774
GZ36	Duyun, Guizhou	E107.5,N26.21	Grassy			470.
GZ37	Fuquan, Guizhou	E107.53,N26.7	Grassy	MaGZ3701	*Metarhizium anisopliae*	471. MH483775
				PlGZ3702	*Purpureocillium lilacinum*	472. MH483776
			Arable	IfGZ3703	*Isaria fumosorosea*	473. MH483777
			orchard	PsGZ3704	*Paecilomyces sp.*	474.
GZ38	Weng'an, Guizhou	E107.41,N27.1	Grassy			475.
GZ39	Kaiyang, Guizhou	E107.08,N26.9	forest	PsGZ3901	*Paecilomyces sp.*	476.
GZ40	Qingzhen, Guizhou	E106.44,N26.74	forest			477.
				L.GZ4002	*Lecanicillium coprophilum*	478. MH483780
GZ41	Dafang, Guizhou	E105.77,N27.12	forest	MaGZ4101	*Metarhizium anisopliae*	479. MH483781
GZ42	Qixingguan, Guizhou	E105.31,N27.25	Grassy	PsGZ4201	*Paecilomyces sp.*	480.
				PmGZ4202	*Metarhizium marquandii*	481. MH483783

Sample NO.	Sample Location	Longitude and Latitude	Soil Vegetation	Isolate NO	Species	Genebank Access NO.
				PsGZ4203	*Purpureocillium sp.*	482.
			orchard	MfGZ4204	*Metarhizium flavoviride*	483. MH483785
				MaGZ4205	*Metarhizium anisopliae*	484. MH483786
			Arable	IfGZ4206	*Isaria fumosorosea*	485. MH483787
				MaGZ4207	*Metarhizium anisopliae*	486. MH483788
GZ43	Dafang, Guizhou	E105.82,N27.27	forest	PmGZ4301	*Metarhizium marquandii*	487. MH483789
				MaGZ4302	*Metarhizium anisopliae*	488. MH483790
				IfGZ4304	*Isaria fumosorosea*	489. MH483791
			orchard	MfGZ4305	*Metarhizium flavoviride*	490. MH483792
			Arable	PsGZ4306	*Paecilomyces sp.*	491.
GZ44	Bozhou, Guizhou	E106.37,N27.52	orchard	PlGZ4402	*Purpureocillium lavendulum*	492. MH483794
				PmGZ4403	*Metarhizium marquandii*	493. MH483795
			forest	PlGZ4404	*Purpureocillium lavendulum*	494. MH483796
GZ45	Renhuai, Guizhou	E106.4,N27.78	crop	MfGZ4501	*Metarhizium flavoviride*	495.
				MaGZ4503	*Metarhizium anisopliae*	496.
GZ46	Bozhou, Guizhou	E106.66,N27.59	Grassy	IfGZ4601	*Isaria javanica*	497. MH483800
				PlGZ4603	*Purpureocillium lavendulum*	498. MH483801
				PmGZ4605	*Metarhizium marquandii*	499. MH483802
			Arable	MaGZ4606	*Metarhizium anisopliae*	500. MH483803
GZ47	Bozhou, Guizhou	E107.15,N27.66	Grassy	L.GZ4702	*Lecanicillium coprophilum*	501. MH483804
GZ48	Meitan, Guizhou	E107.52,N27.75	Grassy	L.GZ4802	*Lecanicillium coprophilum*	502. MH483805
GZ49	Yuqing, Guizhou	E107.91,N27.25	orchard	PlGZ4902	*Purpureocillium lavendulum*	503. MH483806
				PsGZ4903	*Purpureocillium sp.*	504.
				L.GZ4904	*Lecanicillium coprophilum*	505. MH483808
			forest	MaGZ4905	*Metarhizium anisopliae*	506.
GZ50	Zhenyuan, Guizhou	E108.23,N27.35	forest	PmGZ5001	*Metarhizium marquandii*	507.
				BbGZ5002	*Beauveria bassiana*	508.
			orchard	PlGZ5004	*Purpureocillium lavendulum*	509. MH483813
GZ51	Zhenyuan, Guizhou	E108.39,N27.04	Grassy			510.
GZ52	Shiqian, Guizhou	E108.22,N27.49	Arable	MaGZ5201	*Metarhizium anisopliae*	511. MH483814

Sample NO.	Sample Location	Longitude and Latitude	Soil Vegetation	Isolate NO	Species	Genebank Access NO.
				PlGZ5202	*Purpureocillium lilacinum*	512. MH483815
			crop	PmGZ5203	*Metarhizium marquandii*	513. MH483816
GZ53	Sinan, Guizhou	E108.23,N27.73	Arable	L.GZ5303	*Lecanicillium coprophilum*	514.
GZ54	Sinan, Guizhou	E108.23,N27.94	Arable			515.
GZ55	Dejiang, Guizhou	E108.16,N28.34	Grassy	L.GZ5503	*Lecanicillium coprophilum*	516. MH483817
GZ56	Jiangkou, Guizhou	E108.57,N27.82	Grassy	L.GZ5601	*Lecanicillium coprophilum*	517. MH483818
GZ57	Jiangkou, Guizhou	E108.94,N27.72	Grassy	PmGZ5701	*Metarhizium marquandii*	518. MH483819
				L.GZ5702	*Lecanicillium coprophilum*	519. MH483820
			Arable	MaGZ5705	*Metarhizium anisopliae*	520. MH483821
			crop	CsGZ5706	*Cladosporium halotolerans*	521. MH483822
GZ58	Xingyi, Guizhou	E104.95,N25.13	crop	PsGZ5801	*Purpureocillium sp.*	522.
GZ59	Xingyi, Guizhou	E105.03,N24.95	forest			523.
GZ60	Bijiang, Guizhou	E109.2,N27.76	Grassy			524.
GZ91	Panzhou, Guizhou	E104.33,N25.65	forest			525.
GZ92	Pu'an, Guizhou	E105.01,N25.75	crop			526.
GZ93	Guanling, Guizhou	E105.65,N25.98	crop			527.
GZ94	Zhenning, Guizhou	E105.68,N26.02	forest			528.
GZ95	Xingyi, Guizhou	E104.93,N25.03	forest			529.
GZ96	Xixiu, Guizhou	E105.87,N26.21	forest	PlGZ9601	*Purpureocillium lavendulum*	530. MH483824
GZ97	Leishan, Guizhou	E108.18,N26.5	forest			
